# Health state utilities for non small cell lung cancer

**DOI:** 10.1186/1477-7525-6-84

**Published:** 2008-10-21

**Authors:** Beenish Nafees, Megan Stafford, Sonia Gavriel, Shkun Bhalla, Jessamy Watkins

**Affiliations:** 1 United BioSource Corporation, UK, 20 Bloomsbury Square, London, WC1A 2NS, UK; 2 Former employee of Eli Lilly, UK, Lilly House, Priestley Road, Basingstoke, Hampshire, RG24 9NL, UK; 3 Eli Lilly, UK, Lilly House, Priestley Road, Basingstoke, Hampshire, RG24 9NL, UK

## Abstract

**Background:**

Existing reports of utility values for metastatic non-small cell lung cancer (NSCLC) vary quite widely and are not all suitable for use in submissions in the UK. The aim of this study was to elicit UK societal based utility values for different stages of NSCLC and different grade III-IV toxicities commonly associated with chemotherapy treatments. Toxicities included neutropenia, febrile neutropenia, fatigue, diarrhoea, nausea and vomiting, rash and hair loss.

**Methods:**

Existing health state descriptions of metastatic breast cancer were revised to make them suitable as descriptions of metastatic NSCLC patients on second-line treatment. The existing health states were used in cognitive debrief interviews with oncologists (n = 5) and oncology specialist nurses (n = 5). Changes were made as suggested by the clinical experts. The resulting health states (n = 17) were piloted and used in a societal based valuation study (n = 100). Participants rated half of the total health states in a standard gamble interview to derive health state utility scores. Data were analysed using a mixed model analysis.

**Results:**

Each health state described the symptom burden of disease and impact on different levels of functioning (physical, emotional, sexual, and social). The disutility related to each disease state and toxicity was estimated and were combined to give health state values. All disease states and toxicities were independent significant predictors of utility (p < 0.001). Stable disease with no toxicity (our base state) had a utility value of 0.653. Utility scores ranged from 0.673 (responding disease with no toxicity) to 0.473 for progressive disease.

**Conclusion:**

This study reflects the value that society place on the avoidance of disease progression and severe toxicities in NSCLC.

## Background

In 2002, approximately 29,000 people died from lung cancer in England and Wales[1] and it is the most common and the most life-threatening form of cancer in Scotland [2]. Lung cancer is also a major cause of death throughout the rest of the world [3,4].

Non-small cell lung cancer (NSCLC) has a poor prognosis. On average, survival is less than one year [5]. In addition, NSCLC can lead to distressing symptoms such as dyspnea, pain, persistent cough, and loss of appetite [5,6]. Severe symptoms are associated with increased anxiety, loss of functioning and decreased health related quality of life (HRQL) [7-10].

Results of the Big Lung Trial (BLT, Brown et al. [11]) which compared best supportive care with or without chemotherapy by analyzing HRQL data (EORTC QLQ-C30 and LC17, and daily diary cards) demonstrated that there was no large (clinically important) negative effects of chemotherapy on HRQL. Furthermore, no significant differences between groups on physical/emotional functioning, fatigue, dyspenea or pain at 12 weeks were found. In addition, Global HRQL, role functioning, fatigue, appetite loss, and constipation were good indicators of survival at 12 weeks. The sample included patients with stage I or II disease and demonstrates that declining HRQL of patients with NSCLC is largely affected by pain, mobility, functionality, and symptom burden. Chemotherapy provides only modest improvements in survival time however it can lead to severe side effects such as hair loss, nausea, and neutropenia, which may lead people to prefer best supportive care [12,13].

In reviewing the evidence regarding the burden of NSCLC on HRQL it is clear that there is scarce information regarding the preferences of patients or society regarding states of disease. Such information is required in economic evaluations based on cost-utility analysis. Trippoli et al. (2001) [14] report utility and HRQL data (SF-36 and EQ-5D questionnaires) from 95 patients with NSCLC. The results showed that HRQL is significantly worse in metastatic NSCLC patients (physical functioning, p = 0.009; bodily pain, p = 0.016). The mean scores for the 8 domains of the SF-36 ranged from 20.8 (physical role) to 63.0 (social functioning). The EQ-5D mean utility score was 0.58 in the self-classifier and in the visual analogue scale. The authors concluded that HRQL was significantly impaired in NSCLC patients, and more so with metastatic patients.

Lloyd et al. (2005) [15] report societal utility values in metastatic NSCLC using health state descriptions of responding, stable (intravenous (IV) and oral treatment presented separately) disease, progressive disease and a state describing end of life. The health states were validated through interviews with oncologists and nurses. UK societal participants (n = 100) were asked to rate the health states in a standard gamble (SG) interview. Mean SG utility scores ranged from 0.70 (responding disease), to 0.33 (end of life). SG values decreased significantly from responding disease to 'end of life', (F = 32.14, P < 0.0001). However this study did not assess the impact of toxicities.

The present study was designed to adapt existing health state descriptions of metastatic breast cancer [16] to describe patients receiving second-line treatment for NSCLC. Health states developed for a study of metastatic breast cancer were adapted to describe the burden of metastatic NSCLC (progressive disease, stable disease, and responding disease). They included symptom burden and the impact of six grade III – IV toxicities and hair loss associated with second-line treatment. The six grade III – IV toxicities included neutropenia, febrile neutropenia, nausea/vomiting, diarrhoea, rash, and fatigue. Preferences for each health state were elicited from a representative group of members of the general public in the UK.

## Methods

### Development of health states

**Figure 1:**
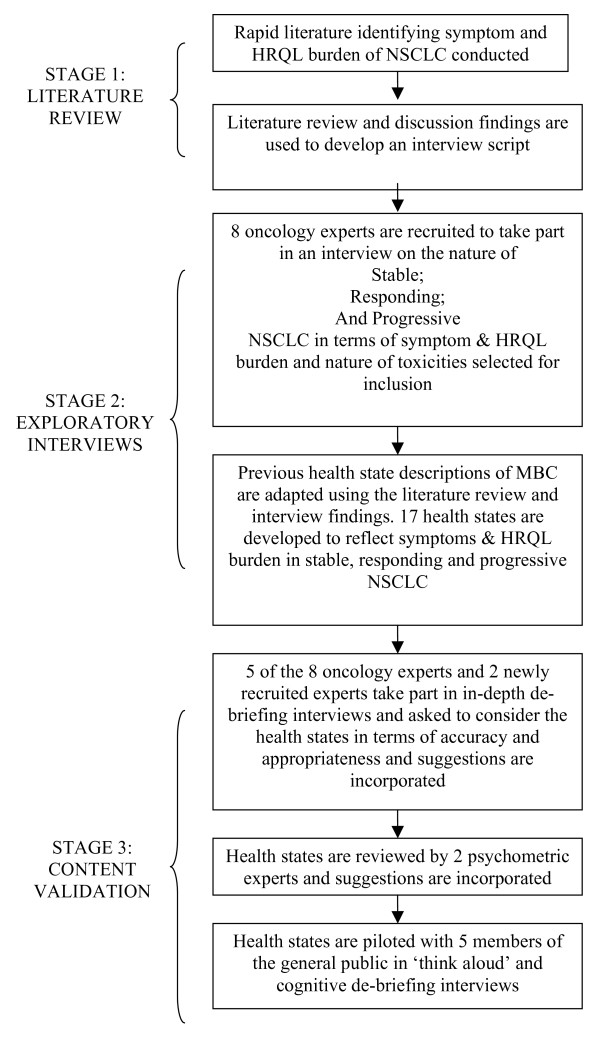
Flow diagram outlining the development of the health states used in the study.

Existing health state descriptions of metastatic breast cancer [16] were used to develop health states to describe patients receiving second-line treatment for metastatic NSCLC. The methodology included a rapid literature review, exploratory interviews with expert physicians and content validation interviews (see figure 1). The health states were produced for a societal valuation study, to be rated by men and women, and were therefore designed to be easily understandable. The health states were designed to describe a three-week period.

### Stage 1 – rapid literature review

A rapid literature review was conducted to understand the nature of symptoms, and HRQL burden in NSCLC. Cancer websites and studies which reported the qualitative nature of symptoms and experience of the disease were sought. Several websites and studies were found which described the severity of symptoms in metastatic NSCLC, and grade III/IV toxicities related to second-line treatment [17-25]. Studies showed that symptoms of disease and chemotherapy-related toxicities had an impact on four main areas of functioning (social, physical, sexual functioning, and emotional wellbeing). The findings were used to develop a standard interview script.

The toxicities were selected because they occurred in over 6% of patients in data from three phase III trials in NSCLC (pemetrexed and docetaxel) [26,27]. Although pulmonary toxicity occurred in 37% of patients [26], an advisory panel of twelve oncology experts suggested that this toxicity should be excluded. The panel advised that pulmonary is a broad toxicity which encompasses many other toxicities and therefore it would be difficult to evaluate. Hair loss was included because it was found to be an important concern to women receiving chemotherapy treatment [28].

### Stage 2 – exploratory interviews

Expert oncology experts were recruited specifically for stage 2 of this developmental process. This included expert physicians (N = 4) and oncology specialist nurses (N = 4) who were identified via an online database of UK medical specialists. These experts took part in telephone interviews conducted by trained interviewers at UBC. Interviews were recorded and transcribed by a professional transcription agency.

The standard interview script was used to guide the interviews. Interviews sought to gain an accurate understanding of the nature of stable, responding and progressive NSCLC in terms of symptom burden, patient experience, the nature and burden of the six toxicities and hair loss, and the impact on four areas of functioning: social, sexual, physical and emotional. Experts were asked to describe patient burden in three base-line disease states: stable (no change in tumour volume), responding (50% reduction in patient's five largest tumours), and progressive (25% growth in patients five largest tumours) disease.

Clinicians described fatigue, breathlessness, cough, and loss of appetite as the most significant symptoms that patients suffer from. These symptoms would be most severe in progressive patients. Each area of functioning included sub areas which addressed particular difficulties that patients might experience, for example physical functioning was examined using four domains of functionality: self-care, caring for your environment, shopping/outdoor duties, and ability to work. Clinicians reported that patients' functionality varied with disease stage. Patients with responding disease were reported as able to do most tasks, whilst patients with stable disease would find it difficult to go out shopping and do daily activities. Patients with progressive disease would not be able to manage most tasks and would 'need assistance with personal hygiene'.

### Stage 3 – content validation

#### (i) Health states – first draft

The literature review and information obtained from interviews with physicians were used to guide the adaptation of health states previously developed for metastatic breast cancer.

Clinicians were queried about the severity and impact of each of the treatment related toxicities in turn in stable and responding disease states. Clinicians stated that all patients at any stage will experience the toxicities, however the tolerance of toxicities may vary with disease stage. Consequently, patients with responding disease would tolerate the toxicities far better than patients with stable or progressive disease. A good consensus emerged from the experts regarding the symptom burden, the nature of toxicities and the impact on areas of functioning.

Seventeen health states were developed to reflect the symptom burden that patients experience in stable, responding and progressive NSCLC, and the nature and burden of toxicities and hair loss associated with second-line treatment in stable and responding disease. Each bullet point in one health state described symptoms of disease and one area of functioning.

#### (ii) Health states validation – interviews

In-depth telephone de-briefing interviews were conducted with some of the original experts (N = 3 physicians and N = 2 nurses) and some new experts (N = 1 physician and N = 1 specialist nurse) to check the accuracy and appropriateness of the newly developed health states. Each health state was examined in terms of its accuracy in depicting HRQL, symptom burden and treatment-related toxicity burden between varying stages of NSCLC.

All interviews were fully transcribed by a professional transcription company. Transcripts were analysed by three researchers independently. Minor changes relating to the wording of the health states were made, but overall clinical experts agreed that the health states were accurate reflections.

Finally, the health states were reviewed by two psychometric experts with expertise in developing measures of HRQL and health states. Their comments and suggestions reflected those made by the physicians and nurses and were incorporated into the health state descriptions.

The health states were piloted with six members of the general public in a 'think aloud' and cognitive de-briefing interview conducted by a trained UBC interviewer (MS). The pilot interviews used the VAS technique only and explored any potential problems with the content of the health states, their comprehensibility and any language issues using a cognitive de-briefing script. Changes were made as necessary based on feedback from the cognitive debrief. The changes involved clarification of terms used to describe neutropenia. The wording was changed to describe the toxicity in everyday language. All the health state descriptions used in this study can be found in appendix 1.

### Main study

Members of the general public were recruited through advertisements in local London newspapers and from an existing UBC database of willing survey participants. One hundred participants who met the eligibility requirements were scheduled for an interview at the UBC offices. The purpose of the interview was fully explained to the participants. Participants were asked to complete a written consent form, and complete a socio-demographic questionnaire and the EQ-5D to rate their own, current health. Trained UBC research interviewers conducted the face-to-face utility interviews. Following their participation in the study all participants received £25 to thank them for taking part.

The interview included two tasks: the visual analogue scale (VAS) and Standard Gamble (SG) utility methods [29,30], which sought to establish people's preferences for health states associated with treatment-related treatment for stable, responding and progressive NSCLC.

The VAS introduces the participant to the concept of rating health states and was conducted prior to the elicitation of the health utilities. The VAS exercise requires the participant to rate each health state on a scale of 0 to 100, where 0 is anchored by 'immediate death' and 100 is anchored by 'perfect health'. The participant rates each health state one by one, and is permitted to alter ratings until satisfied that the relative and absolute ratings accurately reflect his/her preference. Each participant was asked to rate twelve health state cards, including cards describing 'own health' and 'worst health'.

Health state utilities were obtained using the SG approach [29,30]. In the SG task for each health state, patients were asked to choose one of three options: (1) to live in the hypothetical health state with certainty for a pre-defined period of time (in this study, 8 months); (2) to choose between various probabilities of having either *full health *or *worst health *for the next 8 months; or (3) to indicate that the two previous options were equal. Probabilities for option 2 (full health and worst health), were varied sequentially until the patient was indifferent between them. Finally, the worst health state was assessed, based on a gamble between *full health *and *dead*. The utility value for the *worst health *state was determined against *dead *and this was used to recalibrate the values for the other health states on the dead (0) to full health (1) scale.

The study included 19 health states altogether (17 health states developed, 1 own health and 1 worst health, see appendix 1). In order to prevent cognitive burden and possibly affecting participant's performance, the health states were randomized into two selections (A and B) of progressive, stable and responding health states and toxicities. All participants were randomly assigned to rate the following: stable and responding disease with no side effects, progressive disease (anchor states), and half of the remaining states (each toxicity was reviewed in either responding or stable stage), as well as own current health and worst health. Participants therefore rated 12 health states each.

### Statistical analysis

The study aimed to collect data from 100 participants. This was not determined by a formal power analysis partly because there was no specific hypothesis to test. Moreover, a previous study of a similar study design conducted in a sample of 100 members of the general public in the UK demonstrated a low variance (SD range = 0.22–0.29) across utilities for all disease states for metastatic breast cancer, thus suggesting that a larger sample size was not required [16]. The demographic and EQ-5D data were summarised and compared to the UK population. The 2001 national census data for England & Wales [31] was used to compare the demographics of the study participants. In addition the EQ-5D data were summarised to determine how closely the sample matched a previous national survey of health in terms of health status and HRQL [32]. Percentages of the sample reporting moderate or extreme problems on each dimension of the EQ-5D were compared to results of the UK National Survey.

The health state valuations from the SG interview were analysed using a mixed model analysis with random effects on the participant level to determine the change in utility score associated with moving between stages of disease and from no toxicity to one of the toxicities included. The raw data were transformed using a logistic transformation (transformed utility = log ((1-utility)/utility)). It was necessary to transform the health state values in order to place them on a 0 to 1 scale (i.e. best outcome is set to 1 and death is 0). This was done in order to obtain a normal distribution suitable to be used in a standard regression model. The method of restricted maximum likelihood (REML) was used to estimate the repeated measurement model. This was done using a mixed model with random effects at the patient level. The model was parameterized using a "saturated" model with parameters for each of the health states and study covariates such as age, gender and own health measured using the EQ-5D total score. First the model was fitted with all variables, and gradually non-significant parameters were removed on the basis of Akaike Information Criteria and the likelihood ratio test. The most parsimonious model was selected. The final model specification was a fixed effect repeated measurement model with an unstructured covariance matrix. All study covariates were excluded in the final model.

## Results

### Participant characteristics

Of 105 respondents, 100 completed the full interview. Five participants did not complete the interview because in the interviewer's opinion they failed to understand the SG task. The demographic profile of the participants was similar to the UK population in terms of age and in that it was predominantly made up of a white population and included a wide representation of ethnic minority groups (Table 1). However, there were a higher proportion of females (38%) and ethnic minorities [Black (14%) and Asian (9%)] in our sample. A large proportion had completed a university education (27%). Apart from these differences the study sample was a fair representation of the general public in England and Wales.

**Table 1:** Demographic profile of study sample

	**Study Sample (N = 100)**	UK Census & ONS* Data 2001–2004
Age Mean (std. dev.)	40.51 (14.91)	38.2

Gender (%female)	38%	51%

**Ethnic Group**		

White	74.0%	92.1%

Black	14.0%	2.0%

Asian	9.0%	4.0%

Other (includes mixed race)	3.0%	1.9%

**Employment Status**		

Full time	29.0%	-

Part time	23.0%	-

Home maker	8.0%	4.6%

Disabled	5.0%	4.2%

Retired	9.0%	9.8%

Student	15.0%	1.9%

Other	11.0%	2.3%

**Education – leaving age**		

No Formal Qualifications	8.0%	-

GCSE/O Levels (16 yrs)	17.0%	-

A Levels (18 yrs)	26.0%	-

Vocational or work based	11.0%	-

University Degree	27.0%	-

Other	11.0%	-

*ONS = UK Office of National Statistics

Participants were also asked to self-report on five EQ-5D dimensions. Our sample showed a similar distribution of HRQL impairments to the national sample reported by Kind et al. (1998) [32]. Overall our sample reported less moderate and extreme problems on all dimensions, with no extreme problems in mobility, self-care and pain/discomfort than in the Kind et al. study [32]. The distributions of moderate problems are fairly similar in self-care, daily activity, pain/discomfort, and anxiety/depression. There was a difference in the distribution of moderate problems in our sample (9.0%) and Kind et al. study [32] (18.3%).

The mean EQ-5D VAS score for own current health was 80.43 (SD = 16.17) and the mean EQ-5D single index score was 0.896 (SD = 0.159). The mean VAS score for own health from the SG interview in the study sample was 83.05 (SD = 15.70).

### Health state utility values

Table 2 shows the estimates and utility decrements for all disease states and toxicities. All disease states and toxicities were independent significant predictors of utility (p < 0.001). All toxicities were associated with a significant decline in utility compared to stable disease with no toxicity, ranging from -0.03248 (rash) (p = 0.007) to -0.09002 (febrile neutropenia) (p = 0.0001).

**Table 2:** Results of the mixed model analysis

**Parameter**	**Parameter Estimate**	**S.E.**	**Degrees of Freedom**	**t-value**	**P**
Intercept	0.6532	0.02223	99	29.39	<0.0001

Progressive	-0.1798	0.02169	99	-8.29	<0.0001

Response	0.0193	0.006556	99	2.94	0.004

Stable	0	.	.	.	.

Neutropenia	-0.08973	0.01543	99	-5.82	<0.0001

Febrile Neutropenia	-0.09002	0.01633	99	-5.51	<0.0001

Fatigue	-0.07346	0.01849	99	-3.97	0.0001

Nausea & vomiting	-0.04802	0.01618	99	-2.97	0.0038

Diarrhoea	-0.0468	0.01553	99	-3.01	0.0033

Hair loss	-0.04495	0.01482	99	-3.03	0.0031

Rash	-0.03248	0.01171	99	-2.77	0.0066

S.E. = standard error

The base health state (stable disease with no toxicity) had a utility value of 0.653. SG utility scores ranged from 0.67 (responding disease with no toxicity) to 0.47 for progressive disease (Table 3). Moving from stable disease to progressive disease was associated with a significant decline in utility (-0.1798, p = 0.0001). The toxicities are compared with the utility values obtained in Lloyd et al. (2006) [16] study Table 3. The mixed model allows a utility value for any combination of disease states and toxicities to be calculated.

**Table 3:** Utility values for all of the disease + toxicity combinations, as compared with the study by Lloyd et al. (2006)[16]

**Variable**	**Utility values**
Progressive	0.473

Responding	0.673

Responding + diarrhoea	0.626

Responding + fatigue	0.599

Responding + febrile neutropenia	0.582

Responding + hair loss	0.628

Responding + nausea/vomiting	0.624

Responding + neutropenia	0.583

Responding + Rash	0.640

Stable + no side effects	0.653

Stable + fatigue	0.580

Stable + febrile neutropenia	0.563

Stable + hair loss	0.608

Stable + nausea/vomiting	0.605

Stable + neutropenia	0.563

Stable + diarrhoea	0.606

Stable + Rash	0.621

## Discussion

This study reports societal preferences or utility values in the UK for health states related to metastatic NSCLC patients on second-line treatment. Health states described stable, responding, and progressive disease and six grade III/IV toxicities and hair loss, associated with second-line treatment. Health state descriptions were developed from interviews with experts in NSCLC, including oncology specialist nurses and oncologists, reviewed by clinical and psychometric experts and piloted on members of the general public.

The utility data reflect the value that the general public places on being in the health states and their perceived severity. Progressive disease was valued as the worst health state with the lowest utility value of 0.473 with a mean utility decrement of 0.1798 from stable disease with no toxicity. Responding disease with no toxicity obtained the highest utility value of 0.673. The utility values of all toxicities also highlighted the severity of the toxicities and the value that general public placed on avoiding them. The decline in utilities associated with each toxicity ranged from 0.090 to 0.032. Febrile neutropenia was considered the worst toxicity (-0.090) whilst rash was given the least importance (-0.032) by members of the general public. This is supported by some of the qualitative responses from participants, whilst rash is severe, it is comparatively preferable than febrile neutropenia which can be life threatening. Febrile neutropenia and neutropenia produced similar utility decrements (0.0900 and 0.897 respectively).

The perceived severity of some toxicities compared with others is supported by research. Paul et al. (2006) [33] conducted a meta-analysis of randomized controlled trials comparing anti-biotic monotherapy (beta-lactams) for febrile neutropenia. Cefepime was associated with higher all-cause mortality at 30 days than other beta-lactams (RR 1.44, 95% CI 1.06–1.94). Adverse events were significantly more frequent with carbapenems. The use of cefepime for febrile neutropenia is associated with increased mortality. This supports febrile neutropenia as being considered the worst toxicity and possibly life-threatening. Studies have shown that specific domains and areas of functioning are considered more important than others [34]. Osoba et al. [34] studied the stated preferences of 400 patients with either breast, colorectal or NSCLC and in either early stages (stage I or II) or late stages (stage III or IV) of cancer. All patients were either receiving or had received chemotherapy treatment. The investigators used a stated-preference instrument which included all functional domains and symptoms of the EORTC Quality of Life questionnaire (QLQ-C30). In the stated preferences questions, physical functioning was the most important area for all patients. In the ranking exercise, patients with NSCLC ranked nausea and vomiting, pain, and emotional functioning as important factors. In late stage NSCLC, 60% of patients wanted to avoid dyspnoea, followed by nausea and vomiting (59%), role functioning (56%), pain (47%) and emotional functioning (40%). In comparison to other cancer patients advanced NSCLC patients also ranked social functioning as more important than physical functioning. This study highlights the different ways that cancer affects people and how each toxicity and stage can be weighted differently. The current study adapted existing health state descriptions of metastatic breast cancer developed in a previous study [16] to describe patients receiving second-line treatment for NSCLC. Lloyd et al. (2006) [16] conducted a societal preference study in which 100 participants completed standard gamble interviews. Utility values were obtained by asking participants to value health state descriptions describing metastatic breast cancer and five grade III/IV toxicities (febrile neutropenia, stomatitis; diarrhoea and vomiting; fatigue; hand-foot syndrome) and hair loss (Table 3) [16].

In comparison to the current study, Lloyd et al. [16] found higher utility values overall in responding and stable disease states and all toxicities. The utility values of responding disease with no toxicity and stable disease with no toxicity were 0.80 and 0.72 respectively. The lower utility values in the current study could be attributed to present health states describing patients who have comparatively progressed and are on second-line therapy. Patients on second-line chemotherapy have less time of survival and have a greater symptom-burden than patients on first-line therapy.

The current utility values can be compared to previous research. Studies have reported utility values of febrile neutropenia with and without hospitalization in metastatic breast cancer ranging from 0.20–0.47 and 0.66 respectively [35,36]. Launois et al. (1996) [36] reported utility value for febrile neutropenia without hospitalisation of 0.66 which is higher than the current value of 0.56 in this study. This could be due to Launois et al.'s study obtaining nurses' preferences rather than societal preferences. However a methodologically comparable study, Lloyd et al. (2005) [15] reported a similar utility value for febrile neutropenia (0.58) in metastatic breast cancer. The higher utility in Lloyd et al. study could be due to the study describing patients on first-line therapy and thus were considered to be at a better stage than the patients described in the current study. Overall, it is difficult to compare previous utility research with the current study as methods of obtaining utility and samples varied.

The current use of mixed model analysis allowed an estimation of utility scores for varied combinations of disease stage and toxicity. This presents a more realistic approach of patients' experience as it is possible they would experience more than one toxicity at one time and also move between stages of disease.

The study faces one significant potential limitation. It would have been possible to collect these data from patients with the relevant disease using a generic measure such as the EQ-5D. People could have completed the EQ-5D as they experienced treatment for metastatic NSCLC with associated side effects. There are several reasons why this approach was not adopted. One of the principal aims of the current study was to estimate the disutility related to grade III – IV toxicities. In order to accurately capture this from patients it would have been necessary to recruit a representative group of patients currently experiencing the toxicity. Given the severity of the toxicities it would have been a significant (and possibly unethical) burden for patients to complete HRQL questionnaires. It would also have been very difficult to recruit a representative group and so therefore we may have underestimated the true burden. To capture reliable utility data for all of the health states in this study (different disease states combined with different toxicities) would have represented a very significant challenge in terms of patient recruitment. Therefore because of the challenges of recruiting sufficient patients, capturing a representative sample (and so not underestimating the HRQL impact) and avoiding a significant burden on people who are very unwell purely for the purposes of research the present methodology was chosen. Health state descriptions were developed from interviews and input from clinicians and psychometric experts. The health state descriptions were developed after rounds of in-depth interviews with oncologists, oncology specialist nurses and psychometric experts.

## Conclusion

The current study presents the value that the general public in the UK places on avoiding progression of disease and second-line treatment-related toxicities. The utility scores show a utility decrement in moving from responding disease with no toxicity (0.67) to stable disease with no toxicity (0.65) to progressive disease (0.47). In addition, there is a significant decline in utility in the experience of all toxicities (febrile neutropenia, neutropenia, fatigue, nausea and vomiting, diarrhoea, rash and hair loss). This study provides unique SG utility data in metastatic NSCLC which has not been explored to date.

## Abbreviations

BLT: Big Lung Trial; EORTC QLQ-C30: European Organization for Research and Treatment of Cancer Quality-of-Life Questionnaire-Cancer-30 items; EORTC QLQ-LC-17: European Organization for Research and Treatment of Cancer Quality-of-Life Questionnaire-Lung Cancer-17 items; EQ-5D: EuroQoL-5 Dimensions; HRQL: Health-related Quality of Life; NSCLC: Non-Small Cell Lung Cancer; SF-36: Short-Form 36 items; SG: Standard Gamble; UBC: United BioSource Corporation; UK: United Kingdom; VAS: Visual Analogue Scale

## Competing interests

Beenish Nafees, Megan Stafford and Sonia Gavriel are employees of United BioSource Corporation who were paid a fixed price by Eli Lilly to design and conduct this study. Shkun Bhalla is a former employee of Eli Lilly. Jessamy Watkins is a current employee of Eli Lilly. There are no other competing interests

## Authors' contributions

BN and MS designed and conducted the study and carried out data collection. SG performed the statistical analysis. BN and SB participated in the structure and helped to draft the manuscript. All authors read and approved the final manuscript.

## Appendix 1

### Health states – NSCLC

#### Stable with no side effects

∘ You have a life threatening illness which is stable on treatment. You are receiving cycles of treatment which require you to go to the outpatient clinic.

∘ You have lost weight and your appetite is reduced. You sometimes experience pain or discomfort in your chest or under your ribs which can be treated with painkillers. You have shortness of breath and breathing can be painful. You have a persistent nagging cough.

∘ You are able to wash and dress yourself and do jobs around the home. Shopping and daily activities take more effort than usual.

∘ You are able to visit family and friends but often have to cut it short because you get tired.

∘ You sometimes feel less physically attractive than you used to. Your illness has affected your sex drive.

∘ You worry about dying and how your loved ones will cope.

#### Stable with Neutropenia

∘ You have a life threatening illness which is stable on treatment. You are receiving cycles of treatment which require you to go to the outpatient clinic.

∘ You have a blood disorder which leaves you at risk of infection. You may experience fatigue, muscle aches and interrupted sleep. You are at risk of it happening again following your next cycle of treatment.

∘ You have lost weight and your appetite is reduced. You sometimes experience pain or discomfort in your chest or under your ribs which can be treated with painkillers. You have shortness of breath and breathing can be painful. You have a persistent nagging cough.

∘ You have difficulty washing and dressing yourself and doing jobs around the home. You are unable to go shopping and do your usual daily activities.

∘ You don't visit family and friends often because of the risk of infection.

∘ You sometimes feel less physically attractive than you used to. Your illness has affected your sex drive.

∘ You worry about dying and how your loved ones will cope.

#### Stable with Febrile neutropenia

∘ You have a life threatening illness which is stable on treatment. You are receiving cycles of treatment which require you to go to the outpatient clinic.

∘ You had a blood disorder which led to your being hospitalised for about 5 days with a fever and severe flu like symptoms. You received treatment because this blood disorder could have caused you to die within a few days of onset. You are at risk of it happening again following your next cycle of treatment.

∘ You have lost weight and your appetite is reduced. You sometimes experience pain or discomfort in your chest or under your ribs which can be treated with painkillers. You have shortness of breath and breathing can be painful. You have a persistent nagging cough.

∘ You are able to wash and dress yourself with assistance. You are unable to go shopping and do your usual daily activities.

∘ You are not able to visit family and friends.

∘ You sometimes feel less physically attractive than you used to. Your illness has affected your sex drive.

∘ You worry about dying and how your loved ones will cope.

#### Stable with Fatigue

∘ You have a life threatening illness which is stable on treatment. You are receiving cycles of treatment which require you to go to the outpatient clinic.

∘ You often feel extremely tired and weak all over. Your tiredness is not relieved by rest. Most of the time you are frustrated by being too tired to do the things you used to do easily.

∘ You have lost weight and your appetite is reduced. You sometimes experience pain or discomfort in your chest or under your ribs which can be treated with painkillers. You have shortness of breath and breathing can be painful. You have a persistent nagging cough.

∘ You are able to wash and dress yourself. You cannot do jobs around the home, go shopping or do other daily activities because you are too tired.

∘ You are not able to visit family and friends because you are too tired.

∘ You sometimes feel less physically attractive than you used to. Your illness has affected your sex drive.

∘ You worry about dying and how your loved ones will cope.

#### Stable with Nausea and Vomiting

∘ You have a life threatening illness which is stable on treatment. You are receiving cycles of treatment which require you to go to the outpatient clinic.

∘ You recently had a bout of sickness that lasted 2–3 days. During those days, you felt very sick (nausea) and were vomiting intermittently. You received treatment for the vomiting and to re-hydrate you. You are at risk of it happening again following your next cycle of treatment.

∘ You have lost weight and your appetite is reduced. You sometimes experience pain or discomfort in your chest or under your ribs which can be treated with painkillers. You have shortness of breath and breathing can be painful. You have a persistent nagging cough.

∘ You are able to wash and dress yourself and do jobs around the home. Shopping and daily activities take more effort than usual. You were unable to do these things when you had the sickness.

∘ You are able to visit family and friends but often have to cut it short because you get tired. You were unable to visit when you had the sickness.

∘ You sometimes feel less physically attractive than you used to. Your illness has affected your sex drive.

∘ You worry about dying and how your loved ones will cope.

#### Stable with Diarrhoea

∘ You have a life threatening illness which is stable on treatment. You are receiving cycles of treatment which require you to go to the outpatient clinic.

∘ You recently had frequent severe diarrhoea. You were taking treatment for the diarrhoea and to re-hydrate you. You are at risk of it happening again following your next cycle of treatment.

∘ You have lost more weight and your appetite is reduced. You sometimes experience significant pain or discomfort in your chest or under your ribs which can be treated with painkillers. You have shortness of breath and breathing can be painful. You have a persistent nagging cough.

∘ You are able to wash and dress yourself and do jobs around the home. Shopping and daily activities take more effort than usual. You were unable to do these things when you had the diarrhoea.

∘ You are able to visit family and friends but often have to cut it short because you get tired. You were unable to visit when you had the diarrhoea.

∘ You sometimes feel less physically attractive than you used to. Your illness has affected your sex drive.

∘ You worry about dying and how your loved ones will cope.

#### Stable with Hair loss

∘ You have a life threatening illness which is stable on treatment. You are receiving cycles of treatment which require you to go to the outpatient clinic.

∘ You have lost all of your hair including bodily hair.

∘ You have lost weight and your appetite is reduced. You sometimes experience pain or discomfort in your chest or under your ribs which can be treated with painkillers. You have shortness of breath and breathing can be painful. You have a persistent nagging cough.

∘ You are able to wash and dress yourself and do jobs around the home. Shopping and daily activities take more effort than usual.

∘ You are able to visit family and friends but often have to cut it short because you get tired. You feel uncomfortable going out due to your hair loss.

∘ You feel much less physically attractive than you used to. Your illness has affected your sex drive.

∘ You worry about dying and how your loved ones will cope.

#### Stable with Rash

∘ You have a life threatening illness which is stable on treatment. You are receiving cycles of treatment which require you to go to the outpatient clinic.

∘ You have a rash which causes redness and peeling of the skin which is sometimes itchy. You are taking medication to treat this rash. You have a higher risk of infection and it may appear again following your next cycle of treatment.

∘ You have lost weight and your appetite is reduced. You sometimes experience pain or discomfort in your chest or under your ribs which can be treated with painkillers. You have shortness of breath and breathing can be painful. You have a persistent nagging cough.

∘ Someone helps you to wash and dress yourself. You can do jobs around the home. Shopping and daily activities take more effort than usual.

∘ You are able to visit family and friends but often have to cut it short because you get tired. You felt uncomfortable going out while you had the rash.

∘ You feel much less physically attractive than you used to. Your illness has affected your sex drive.

∘ You worry about dying and how your loved ones will cope.

#### Responding with no side effects

∘ You have a life threatening illness which is responding to treatment. You are receiving cycles of treatment which require you to go to the outpatient clinic.

∘ You are gaining back your weight and your appetite is returning. You occasionally experience pain or discomfort in your chest or under your ribs which can be treated with painkillers. You sometimes have shortness of breath. You occasionally have a nagging cough.

∘ You are able to wash and dress yourself and do jobs around the home. Shopping and daily activities can sometimes be tiring.

∘ You are able to visit family and friends but sometimes have to cut it short because you get tired.

∘ You occasionally feel less physically attractive than you used to. Your illness has somewhat affected your sex drive.

∘ You sometimes worry about dying and how your loved ones will cope.

#### Responding with Neutropenia

∘ You have a life threatening illness which is responding on treatment. You are receiving cycles of treatment which require you to go to the outpatient clinic.

∘ You have a blood disorder which leaves you at risk of infection. You may experience fatigue, muscle aches and interrupted sleep. You are at risk of it happening again following your next cycle of treatment.

∘ You are gaining back your weight and your appetite is returning. You occasionally experience pain or discomfort in your chest or under your ribs which can be treated with painkillers. You sometimes have shortness of breath. You occasionally have a nagging cough.

∘ You have difficulty washing and dressing yourself and doing jobs around the home. You are unable to go shopping and do your usual daily activities.

∘ You don't visit family and friends often because of the risk of infection.

∘ You sometimes feel less physically attractive than you used to. Your illness has somewhat affected your sex drive.

∘ You sometimes worry about dying and how your loved ones will cope.

#### Responding with Febrile Neutropenia

∘ You have a life threatening illness which is responding to treatment. You are receiving cycles of treatment which require you to go to the outpatient clinic.

∘ You had a blood disorder which led to your being hospitalised for about 5 days with a fever and severe flu like symptoms. You received treatment because this blood disorder could have caused you to die within a few days of onset. You are at risk of it happening again following your next cycle of treatment.

∘ You are gaining back your weight and your appetite is returning. You occasionally experience pain or discomfort in your chest or under your ribs which can be treated with painkillers. You sometimes have shortness of breath. You occasionally have a nagging cough.

∘ You are able to wash and dress yourself with assistance. You are unable to go shopping and do your usual daily activities.

∘ You are not able to visit family and friends.

∘ You occasionally feel less physically attractive than you used to. Your illness has somewhat affected your sex drive.

∘ You sometimes worry about dying and how your loved ones will cope.

#### Responding with Fatigue

∘ You have a life threatening illness which is responding to treatment. You are receiving cycles of treatment which require you to go to the outpatient clinic.

∘ You feel extremely tired and weak all over. Your tiredness is not relieved by rest. Most of the time you are frustrated by being too tired to do the things you used to do easily.

∘ You are gaining back your weight and your appetite is returning. You occasionally experience pain or discomfort in your chest or under your ribs which can be treated with painkillers. You sometimes have shortness of breath. You occasionally have a nagging cough.

∘ You are able to wash and dress yourself. You cannot do jobs around the home, shopping or other daily activities because you are too tired.

∘ You are not able to visit family and friends because you are too tired.

∘ You occasionally feel less physically attractive than you used to. Your illness has somewhat affected your sex drive.

∘ You sometimes worry about dying and how your loved ones will cope.

#### Responding with Nausea and Vomiting

∘ You have a life threatening illness which is responding to treatment. You are receiving cycles of treatment which require you to go to the outpatient clinic.

∘ You recently had a bout of sickness that lasted 2–3 days. During those days, you felt very sick (nausea) and were vomiting intermittently. You received treatment for the vomiting and to re-hydrate you. You are at risk of it happening again following your next cycle of treatment.

∘ You are gaining back your weight and your appetite is returning. You occasionally experience pain or discomfort in your chest or under your ribs which can be treated with painkillers. You sometimes have shortness of breath. You occasionally have a nagging cough.

∘ You are able to wash and dress yourself and do jobs around the home. Shopping and daily activities take more effort than usual. You were unable to do these things when you had the sickness.

∘ You are able to visit family and friends but sometimes have to cut it short because you get tired. You were unable to visit when you had the sickness.

∘ You occasionally feel less physically attractive than you used to. Your illness has somewhat affected your sex drive.

∘ You sometimes worry about dying and how your loved ones will cope.

#### Responding with Diarrhoea

∘ You have a life threatening illness which is responding to treatment. You are receiving cycles of treatment which require you to go to the outpatient clinic.

∘ You recently had frequent severe diarrhoea. You were taking treatment for the diarrhoea and to re-hydrate you. You are at risk of it happening again following your next cycle of treatment.

∘ You have lost some weight and your appetite is returning. You lost some weight when you had severe diarrhoea. You occasionally experience pain or discomfort in your chest or under your ribs which can be treated with painkillers. You sometimes have shortness of breath. You occasionally have a nagging cough.

∘ You are able to wash and dress yourself and do jobs around the home with difficulty. You are unable to go shopping and do your usual daily activities because of the diarrhoea.

∘ You are able to visit family and friends but sometimes have to cut it short because you get tired. You were unable to visit when you had the diarrhoea.

∘ You occasionally feel less physically attractive than you used to. Your illness has somewhat affected your sex drive.

∘ You sometimes worry about dying and how your loved ones will cope.

#### Responding with Hair loss

∘ You have a life threatening illness which is responding to treatment. You are receiving cycles of treatment which require you to go to the outpatient clinic.

∘ You have lost all of your hair including bodily hair.

∘ You are gaining back your weight and your appetite is returning. You occasionally experience pain or discomfort in your chest or under your ribs which can be treated with painkillers. You sometimes have shortness of breath. You occasionally have a nagging cough.

∘ You are able to wash and dress yourself and do jobs around the home. Shopping and daily activities can sometimes be tiring.

∘ You are able to visit family and friends but sometimes have to cut it short because you get tired. You feel uncomfortable going out due to your hair loss.

∘ You sometimes feel much less physically attractive than you used to. Your illness has somewhat affected your sex drive.

∘ You sometimes worry about dying and how your loved ones will cope.

#### Responding Rash

∘ You have a life threatening illness which is responding on treatment. You are receiving cycles of treatment which require you to go to the outpatient clinic.

∘ You have a rash which causes redness and peeling of the skin which is sometimes itchy. You are taking medication to treat this rash. You have a higher risk of infection and it may appear again following your next cycle of treatment.

∘ You are gaining back your weight and your appetite is returning. You sometimes experience pain or discomfort in your chest or under your ribs which can be treated with painkillers. You sometimes have shortness of breath. You occasionally have a nagging cough.

∘ You are able to wash and dress yourself and do jobs around the home. Shopping and daily activities can sometimes be tiring.

∘ You are able to visit family and friends but sometimes have to cut it short because you get tired. You felt uncomfortable going out while you had the rash.

∘ You sometimes feel much less physically attractive than you used to. Your illness has somewhat affected your sex drive.

∘ You sometimes worry about dying and how your loved ones will cope

#### Progressive

∘ You have a life threatening illness and your condition is getting worse.

∘ You have lost your appetite and have experienced significant weight loss. You experience pain and discomfort in your chest or under your ribs. You frequently have shortness of breath and breathing is often painful. You have a persistent nagging cough and sometimes cough up blood. You may experience some difficulty swallowing.

∘ You experience severe fatigue and feel too tired to go out or to see family and friends. It has affected your relationships with them.

∘ You need assistance to wash and dress yourself. You are often unable to do jobs around the house or other daily activities. You are dependent on others to do your shopping and are unable to do your usual daily activities.

∘ You often feel less physically attractive than you used to. You have little or no sexual drive.

∘ You're depressed and dying is always on your mind. You worry about how your loved ones will cope.

### Own Health

• Think about your own current health and how you have been feeling.

• Think about how your health and medical treatment affect areas of your life, including:

- Employment

- Ability to care for yourself

- Ability to take part in your regular activities

- How you feel emotionally

- The amount of pain, discomfort and symptoms you experience.

### Worst Health

• Your condition is critical. You are receiving strong medication to relieve any increasing pain and nausea but it is not helping. You experience constant severe fatigue. You hardly eat and you have lost a large amount of weight.

• You are too tired to see family and friends and conversing with visitors has become difficult.

• You may have been moved to a palliative care unit or be receiving hospice service at home. You are confined to a bed or chair, and need assistance using the toilet, washing, dressing and eating etc.

• Your gaunt, frail appearance is alarming to yourself and your family.

• You feel a burden on loved ones and your lack of independence is demoralizing. You have lost hope for recovery. You are afraid of dying in pain and have begun discussing your concerns with your caregivers.

## References

[B1] NICE (2005). Lung cancer: diagnosis and treatment.

[B2] Scottish Executive Health Department (2001). Cancer scenarios: an aid to planning cancer services in Scotland in the next decade.

[B3] Parkin DM, Bray FI, Devessa SS (2001). Cancer burden for the year 2000: The global picture. Eur J Cancer.

[B4] Jemal A, Murray T, Samuels A, Ghafoor A, Ward E, Thun MJ (2003). Cancer statistics, 2003. CA Cancer J Clin.

[B5] Busick NP, Fretz PC, Galvin JR, Peterson MW (1999). Staging of small cell and non-small cell lung cancer.

[B6] Hopwood P, Stephens RJ (1995). Symptoms at presentation for treatment in patients with lung cancer: Implications for the evaluation of palliative treatment. BR J Cancer.

[B7] Krech RL, Davis J, Walsh D, Curtis EB (1992). Symptoms of lung cancer. Palliat Med.

[B8] Vainio A, Auvinen A, with members of the Symptom prevalence Group (1996). Prevalence of symptoms among patients with advanced cancer: An international collaborative study. J Pain Symptom Manage.

[B9] Handy JR, Asaph JW, Skokan L, Reed CE, Koh S, Brooks G, Douville EC, Tsen AC, Ott GY, Silvestri GA (2002). What happens to patients undergoing lung cancer surgery? Outcomes and quality of life before and after surgery. Chest.

[B10] Cooley ME, Short TH, Moriarty HJ (2003). Symptom Prevalence, distress, and change over time in adults receiving treatment for lung cancer. Psycho-oncology.

[B11] Brown J, Thorpe H, Napp V, Fairlamb DJ, Gower NH, Milroy R, Parmar MKB, Rudd RM, Spiro SG, Stephens RJ, Waller D, West P, Peake MD (2005). Assessment of Quality of Life in the Supportive Care setting of the Big Lung Trial in Non-Small-Cell Lung cancer. Journal of Clinical Oncology.

[B12] Silvestri G, Pritchard R, Welch HG (1998). Preferences for chemotherapy in patients with advanced non-small cell lung cancer: descriptive study based on scripted interviews. BMJ.

[B13] Socinski MA, Morris DE, Masters GA, Lilenbaum R (2003). Chemotherapeutic management of stage IV non-small cell lung cancer. Chest.

[B14] Trippoli S, Vaiani M, Lucioni C, Messori A (2001). Quality of Life and utility in patients with non-small cell lung cancer. Quality of Life Study Group of the Master 2 Project in Pharmacoeconomics. Pharmacoeconomics.

[B15] Lloyd A, van Hanswijck de Jonge P, Doyle S, Walker M, Farina C (2005). Development and elicitation of health state utilities in Metastatic Non Small cell lung cancer (NSCLC) in the UK. Poster presented at: The 27th Annual Meeting of the Society for Medical Decision Making.

[B16] Lloyd A, Nafees B, Narewska J, Dewilde S, Watkins J (2006). Health state utilities for metastatic breast cancer. Br J Cancer.

[B17] Cancer Research UK. http://www.cancerresearchuk.org.

[B18] Cancer Help. http://www.cancerhelp.org.uk.

[B19] Association for International Cancer Research. http://www.aicr.org.uk/.

[B20] DIPEX. http://www.dipex.org.

[B21] Montazeri A, Milroy R, Macbeth FR, McEwen J, Gillis CR (1996). Understanding patients: lets talk about it. Support Care Cancer.

[B22] Fortner BV, Tauer KW, Okon T, Houts AC, Schwartzberg LS (2005). Experiencing neutropenia: Quality of life interviews with adult cancer patients. BMC Nursing.

[B23] Murray SA, Boyd K, Kendall M, Worth A, Benton TF, Clausen H (2002). Dying of lung cancer or cardiac failure: Prospective qualitative interview study of patients and their carers in the community. BMJ.

[B24] Murray SA, Grant E, Grant A, Kendall M (2003). Dying from cancer in developed and developing countries: lessons from two qualitative interview studies of patients and their carers. BMJ.

[B25] (1995). NICE Guidelines.

[B26] Shepherd FA, Dancey J, Ramlau R, Mattson K, Gralla R, O'Rourke M, Levitan N, Gressot L, Vincent M, Burkes R, Coughlin S, Kim Y, Berille J (2000). Prospective randomized trial of docetaxel versus best supportive care in patients with non-small cell lung cancer previsouly treated with platinum based chemotherapy. J Clin Oncol.

[B27] Hanna N, Shepherd FA, Fossella FV, Pereira JR, De Marinis F, von Pawel J, Gatzemeier U, Tsao TC, Pless M, Muller T, Lim HL, Desch C, Szondy K, Gervais R, Shaharyar, Manegold C, Paul S, Paoletti P, Einhorn L, Bunn PA (2004). Randomized phase III trial of pemetrexed versus docetaxel in patients with non-small cell lung cancer previously treated with chemotherapy. J Clin Oncol.

[B28] Hopwood P, Watkins J, Ellis P, Smith I (2004). Clinical interpretation of quality of life outcomes: investigation in a randomized trial of gemcitabine plus paclitaxel compared to paclitaxel alone for metastatic breast cancer (MBC). Annals of Oncology.

[B29] Torrance GW (1986). Measurement of health state utilities for economic appraisal. J Health Econ.

[B30] Bennett KJ, Torrance GW, Spilker B (1996). Measuring health state preferences and utilities: rating scale, time trade-off, and standard gamble techniques. Quality of Life and Pharmacoeconomics in Clinical Trials.

[B31] Office of National Statistics, National Statistics website: (2001) Population of the United Kingdom: by ethnic group. http://www.statistics.gov.uk/cci/nugget_print.asp?ID=764.

[B32] Kind P, Dolan P, Gudex C, Williams A (1998). Variations in population health status: results from a United Kingdom national questionnaire survey. British Medical Journal.

[B33] Paul M, Yahav D, Fraser A, Leobivici L (2006). Empirical antibiotic monotherapy for febrile neutropenia: systematic review and meta-analysis of randomized controlled trials. J Antimicrob Chemotherapy.

[B34] Osoba D, Hsu MA, Copley-Merriman C, Coombs J, Johnson FR, Hauber B, Manjunath R, Pyles A (2006). Stated preferences of patients with cancer for health-related quality-of-life (HRQL) domains during treatment. Qual Life Res.

[B35] Hutton J, Brown R, Borowitz M, Abrams K, Rothman M, Shakespeare A (1996). A new decision model for cost-utility comparisons of chemotherapy in recurrent metastatic breast cancer. Pharmacoeconomics.

[B36] Launois R, Reboul-Marty J, Henry B, Bonneterre J (1996). A cost-utility analysis of second-line chemotherapy in metastatic breast cancer: Docetaxel versus paclitaxel versus vinorelbine. Pharmacoeconomics.

